# Evaluation of Acute and Subacute Toxicities of *Psidium guajava* Methanolic Bark Extract: A Botanical with *In Vitro* Antiproliferative Potential

**DOI:** 10.1155/2019/8306986

**Published:** 2019-12-11

**Authors:** Hermione T. Manekeng, Armelle T. Mbaveng, Samuel A. Ntyam Mendo, Armel-Joseph D. Agokeng, Victor Kuete

**Affiliations:** Department of Biochemistry, Faculty of Science, University of Dschang, Dschang, Cameroon

## Abstract

The methanol crude extract of the bark of *Psidium guajava* (guava) previously displayed interesting cytotoxic effects on a panel of human cancer cell lines. In the present work, we plan to determine the toxicological effects of this guava botanical in Wistar rats. Acute oral toxicity of the extract was carried out by administration of a single dose of 5000 mg/kg body weight to female rats in 14 days. Subacute toxicity was conducted by oral administration of the extract at daily doses of 250 mg/kg, 500 mg/kg, and 1000 mg/kg body weight, respectively, while rats in the control group received no extract. After 28 days of treatment, animals were sacrificed for hematological and biochemical studies. In the acute toxicity study, no mortality or signs of toxicity were recorded; hence, the median lethal dose (LD_50_) of the *Psidium guajava* bark extract is greater than 5000 mg/kg body weight. For the subacute toxicity study, significant variations in body weight, relative weight of organs, and biochemical parameters were observed in the animals treated at different doses of the plant extract compared to control animals. Histopathological analyses showed minor liver inflammation in females treated at the highest dose (1000 mg/kg). These results suggest that intake of a single high dose of the *Psidium guajava* bark extract is nontoxic, but repeat administration could exhibit mild organ toxicity.

## 1. Introduction

Because of its great diversity in medicinal plants, nature gives humanity the gift of an immense therapeutic wealth [1]. The consumption of natural substances to cure and prevent diseases is old and universal; it plays an important role in access to basic health care for populations [2]. Research on the therapeutic potential of plants has flown over the years with a wealth of scientifically proven information, showing the considerable power of plants in the treatment of a wide range of diseases [3]. All parts of the plant that is the subject of this work especially leaves are traditionally recognized in the treatment of diarrhea, dysentery, gastrointestinal problems, gastric pain, respiratory problems, obesity, and hypertension [4, 5].

*Psidium guajava*, commonly known as guava, is a plant of the family Myrtaceae, native to South America, but to date widespread throughout the tropical zone especially where the climate is favorable [6]. This medium-sized plant, up to 10 m tall, is used as food, and all its parts have pharmacological properties [7]. It has a plethora of laboratory-proven biological activities such as antimicrobial [8], antidiarrheal [9], hepatoprotective [10], antioxidant [11], and anticancer [12]. This latter activity has already been the subject of numerous studies, as the work of Salib and Michael [13] demonstrated that the aqueous extract of *P. guajava* leaves inhibits the viability of cancer cell lines DU-145 in a dose-dependent manner, while its essential oil has antiproliferative activity four times more potent than that of vincristine, a reference anticancer drug on P388 lines. Similarly, the methanol extract of the peel of its fruit has promising *in vitro* activities against MCF-7 cells of human breast cancer [14]. The methanolic bark extract of *P. guajava* revealed considerable cytotoxic activity against CEM/ADR5000 cells with a 50% inhibitory concentration (IC50) of 1.29 *μ*g/mL, against HCT 116 cells (p53+/+) with an IC50 of 18.63 *μ*g/mL, and against several other sensitive and resistant cells with IC50 values up to 62.64 *μ*g/mL (towards MDA-MB-231 cells) [12].

However, plants in addition to their effectiveness must be free of harmful side effects for the consumer because the main criterion for the selection of medicinal plants is above all safety [1]. But the literature reveals that existing plant efficacy data are far more abundant than those for toxicity [15]. In this regard, the literature offers insufficient information on the toxicological effects related to the use of *P. guajava* extracts. It is therefore imperative to obtain more toxicological data from this plant in order to ensure its safety vis-à-vis humans and especially for the development of new drugs [16]. Our study aims to evaluate the acute and subacute toxicities of the methanol extract of the *P. guajava* bark in Wistar rats.

## 2. Subjects and Methods

### 2.1. Plant Material

The bark of *P. guajava* used in this work was harvested in West Cameroon (in the locality of Dschang) in February 2018. A sample of this plant was authenticated by Mr. Jumbam Blaise at the National Herbarium of Cameroon (HNC) in Yaoundé compared to the reference sample number 2884/SRF/Cam.

### 2.2. Preparation of the Plant Extract

The plant was cleaned and dried under the sun for a week and ground. The powder obtained was macerated in methanol for 48 hours in the proportion of 1/3. The mixture was then filtered through Whatman No. 1 paper and concentrated using a rotary evaporator (Buchi R-200) at 65°C. The extract obtained was dried in an oven at 40°C to completely evaporate the residual solvent. After complete drying, the extract was weighed and stored in a refrigerator at 4°C.

### 2.3. Experimental Animals

Three nulliparous and nonpregnant female rats of age 12 weeks were used for the acute toxicity study. For the subacute toxicity study, 32 animals weighing between 170 and 245 g (16 males and 16 females) including nulliparous and nonpregnant females were used. All these animals were raised under experimental conditions at the animal house of Department of Biochemistry, University of Dschang. They were kept at room temperature, and the lighting sequence was that offered by the day and night cycles. The animals were individualized one week before the start of the experiment; they ate a standard food [17] and received drinking water *ad libitum*.

### 2.4. Acute Oral Toxicity: Experimental Procedure

The acute toxicity test was conducted according to OECD Test Guideline 425 [18] for acute oral toxicity. Animals were fasted for 12 hours and weighed before undergoing intragastric feeding. They received the *P. guajava* extract at a single dose of 5000 mg/kg b.w. After administration, the animals were deprived again of food for 4 hours during which they were observed individually to note any manifestation of toxicity (reduced mobility, aggressiveness, faeces appearance, and sensitivity to noise and pain). They were then observed daily for 14 days for signs of morbidity or mortality. On the 15th day of the test, animals were anesthetized with chloroform vapor and sacrificed for macroscopic observation of organs (liver, kidney, spleen, heart, and lungs).

### 2.5. Subacute Toxicity

This test was conducted according to OECD Guideline 407 [19]. In brief, 32 rats were randomly divided into 4 groups of 08 animals each (04 males and 04 females). Group 1 rats were used as controls and received daily distilled water as a vehicle, while rats in groups 2, 3, and 4 received the *P. guajava* extract at doses of 250, 500, and 1000 mg/kg b.w., respectively, by intragastric gavage each day for 28 days. During this period, the animals were examined daily for signs of toxicity; their mass and their food consumption were recorded every 02 and 03 days, respectively.

### 2.6. Sacrifice of Animals and Collection of Blood, Urine, and Organs

On the 29th day, after a fast of 12 h, all animals were stressed in order to collect the urine; they were subsequently anesthetized with chloroform vapor, and the blood samples were taken by cardiac puncture in EDTA tubes and without EDTA. The first samples were for the determination of hematological parameters and the second for the analysis of biochemical parameters. The organs (kidney, liver, heart, lung, and spleen) were removed, cleaned with physiological saline, and weighed to calculate their relative weights. Liver and kidney samples from each dose group were preserved in 10% formalin solution for histopathological studies.

### 2.7. Determination of Weight Gain and Relative Weight of Organs

The animal's body weight determined before and at the end of the test made it possible to calculate the weight gain by subtracting the starting body weight from that at the end of the test. Relative weight of organs was calculated using the following formula:

(1) $$\begin{array}{c} \text{Relative weight}=\frac{\text{weight of the organ}}{\text{animal body weight}}\times 100. \end{array}$$

### 2.8. Hematological Parameters

Plasma components such as white blood cells, monocytes, granulocytes, red blood cells, hemoglobin, hematocrit, mean corpuscular volume, mean corpuscular hemoglobin, lymphocytes, and platelets were listed using an automated analyzer hematology.

### 2.9. Biochemical Parameters

Serum activities of AST and ALT, as well as serum levels of total protein, total cholesterol, HDL cholesterol, creatinine, and urea, were determined using specific commercial kits (SGM Italia).

### 2.10. Histopathological Analysis

Liver and kidney samples were stored in 10% formalin and processed by conventional techniques. These tissues were subsequently dehydrated in increasing concentration of alcohol, inserted into paraffin, and cut into sections of 4-5 *μ*m. These paraffin sections (5 *μ*m thick) were stained with hematoxylin-eosin prior to microscopic examination [20].

### 2.11. Statistical Analysis

Results obtained were presented as average ± standard deviation; GraphPad Prism software version 5 was used for one-way analysis of variance followed by the Newman–Keuls multiple comparison test. Values were considered significant at the 5% probability level (*p* < 0.05).

## 3. Results

### 3.1. Acute Oral Toxicity

In this study, no death was recorded following the administration of 5000 mg/kg b.w. of the *P. guajava* methanolic bark extract. The animals were apparently healthy and showed no signs of toxicity at this dose extract. Macroscopic observation of organs (liver, kidney, heart, spleen, and lung) showed no alteration. Thus, the LD_50_ was greater than 5000 mg/kg b.w. Animals' body weight during the test is recorded in Table 1.

### 3.2. Subacute Oral Toxicity

#### 3.2.1. Weight Growth and Food Intake

**Figure 1:**
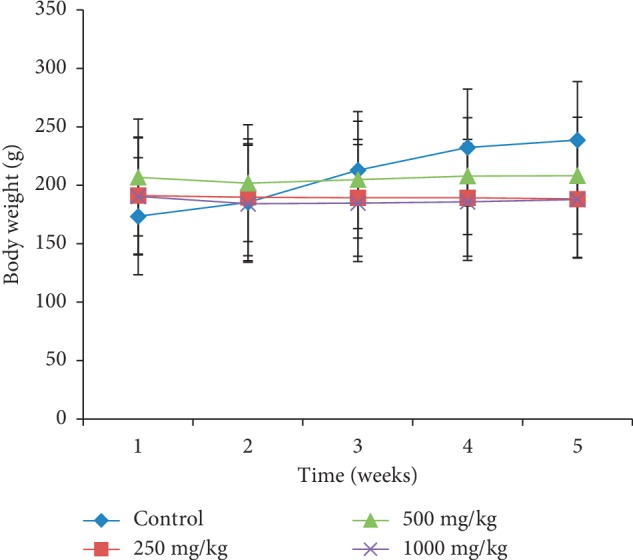
Effect of oral administration of the *Psidium guajava* extract on body weight changes of female rats during 28 days of the subacute toxicity study.

**Figure 2:**
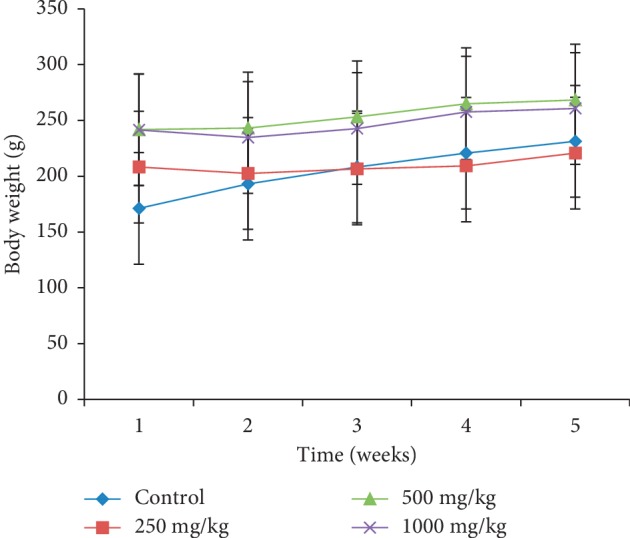
Effect of oral administration of the *Psidium guajava* extract on body weight changes of male rats during 28 days of the subacute toxicity study.

**Figure 3:**
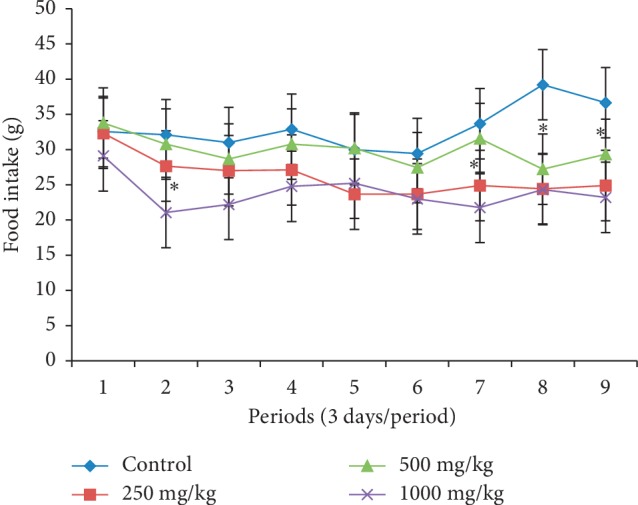
Effect of oral administration of the *Psidium guajava* extract on food intake of female rats during 28 days of the subacute toxicity study. ^*∗*^indicates significant values at *p* < 0.05.

**Figure 4:**
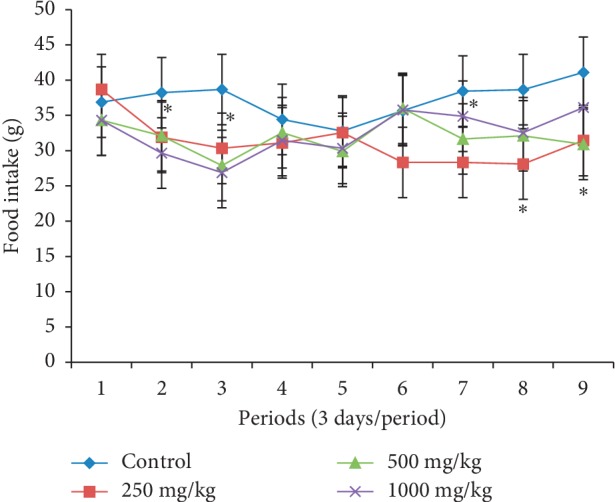
Effect of oral administration of the *Psidium guajava* extract on food intake of male rats during 28 days of the subacute toxicity study. ^*∗*^indicates significant values at *p* < 0.05.

Body weight changes in females treated at different doses of the extract during the four weeks of the study presented an almost constant body weight compared to those in the control whose evolution was growing (Figure 1). On the contrary, in male rats treated with the *P. guajava* methanolic bark extract, body weight slightly increased (Figure 2). All female and male animals that received the plant extract had a significantly lower (*p* < 0.05) food intake (Figures 3 and 4).

#### 3.2.2. Relative Weight of Organs

Relative weight values of organs of all animals are presented in Table 2. In females, the liver and spleen decreased at all extract doses (*p* < 0.001), while the kidney, heart, and lungs significantly increased (*p* < 0.001) in weight compared to those in control rats. In males, decreased weight of the liver, spleen, and lung (all doses, *p* < 0.001) and heart (500 and 1000 mg/kg, *p* < 0.001) of animals treated with the plant extract has been observed; relative weight of the kidney showed no significant difference from that of the control.

#### 3.2.3. Hematological Parameters

Administration of the *P. guajava* methanolic bark extract to animals increases (*p* < 0.05) the red blood cell number and reduces the amount of platelets in females (1000 mg/kg). In males (1000 mg/kg), it resulted in an increase (*p* < 0.01) in the platelet number. However, the other parameters showed no significant difference (*p* > 0.05) at all tested doses compared to those in control animals. All of these data are presented in Table 3.

#### 3.2.4. Urinary Biochemical Parameters

Values of parameters evaluated in the urine of animals are summarized in Table 4. In females, there were a decrease in the creatinine level (all doses, *p* < 0.05) and an increase in protein levels (500 and 1000 mg/kg, *p* < 0.001). However, there was no significant difference (*p* > 0.05) for urea between the treated and control females. In males, no significant difference (*p* > 0.05) was observed for creatinine, while urea (*p* < 0.001) and protein (*p* < 0.01) increased at the doses 500 and 1000 mg/kg b.w.

#### 3.2.5. Serum Biochemical Parameters

Investigations made on the animal's sera made it possible to obtain data in Table 5. In females, there was a decrease in HDL cholesterol levels (500 and 1000 mg/kg, *p* < 0.05), triglycerides (*p* < 0.01), total protein (*p* < 0.001), and creatinine (*p* < 0.05) at all doses. AST levels (*p* < 0.01), total cholesterol (*p* < 0.001), LDL cholesterol (*p* < 0.01), and urea (*p* < 0.01) increased for all doses of the extract compared to those in control animals, while ALT levels showed no significant difference. In males, there was no significant difference between triglyceride levels of the test animals and control animals (*p* > 0.05). Total protein levels (500 and 1000 mg/kg, *p* < 0.001), ALT (*p* < 0.01), AST, total cholesterol, and LDL cholesterol (*p* < 0.001) decreased significantly at all doses, while urea (1000 mg/kg, *p* < 0.01), HDL cholesterol (*p* < 0.01), and creatinine (*p* < 0.001) increased for all extract doses.

#### 3.2.6. Histological Studies

**Figure 5:**
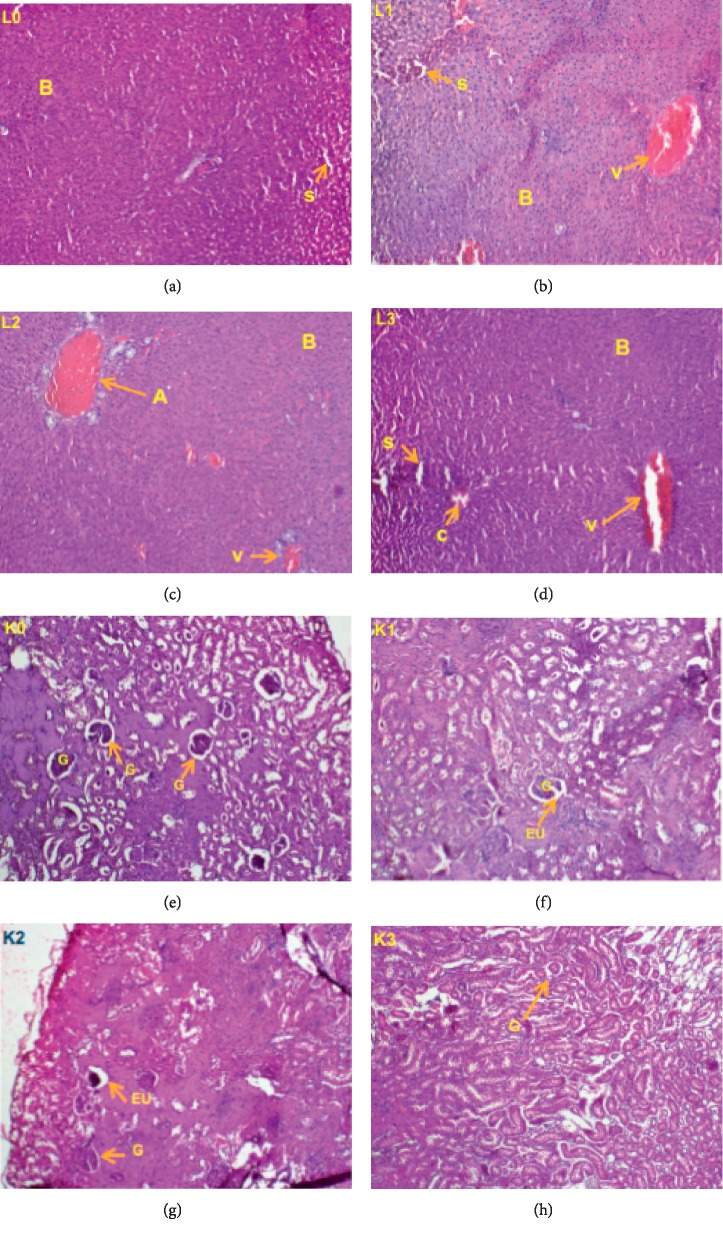
Liver (L) and kidney (K) sections of female rats showing effects of oral administration of the *Psidium guajava* extract over 28 days; L0, K0: control group; L1, K1: 250 mg/kg; L2, K2: 500 mg/kg; L3, K3: 1000 mg/kg. Indicators: A, branch of the hepatic portal vein; B, hepatocytes; c, leukocyte infiltration inflammation; s, sinusoid; v, centrolobular vein; G, glomerulus; EU, urinary space.

**Figure 6:**
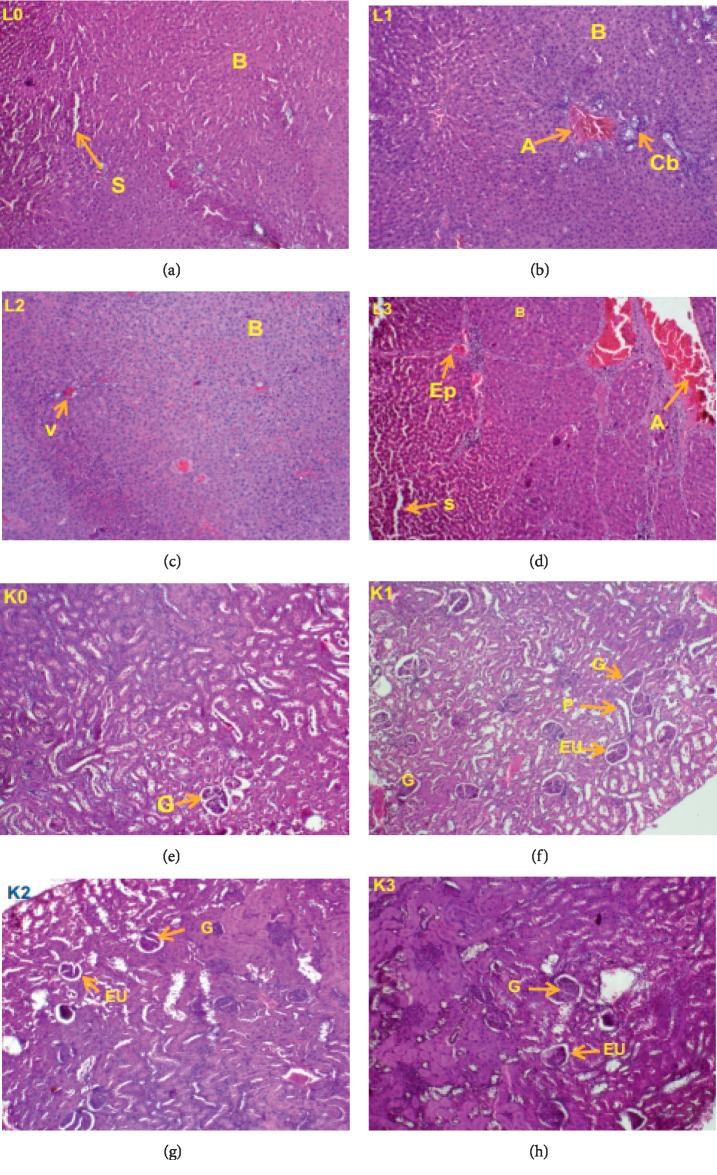
Liver (L) and kidney (K) sections of male rats showing effects of oral administration of the *Psidium guajava* extract over 28 days; L0, K0: control group; L1, K1: 250 mg/kg; L2, K2: 500 mg/kg; L3, K3: 1000 mg/kg. Indicators: A, branch of the hepatic portal vein; B, hepatocytes; s, sinusoid; v, centrolobular vein; G, glomerulus; EU, urinary space; Ep, hepatic portal space; Cb, bile duct; P, proximal convoluted tubule.

Histopathological examinations were performed on the kidney and liver of animals to verify whether they were damaged by the daily administration of the *P. guajava* methanolic bark extract; different observations are illustrated. In females (Figure 5), the liver appeared normal at doses 250 and 500 mg/kg b.w., but at the dose 1000 mg/kg, there was inflammation (infiltration of leucocytes). The kidney, on the contrary, showed no alteration although color changes were observed. However, in male rats (Figure 6), the liver as well as the kidney showed no damage at all dose levels tested.

## 4. Discussion

This study was conducted to prevent human exposure to potential risks associated with the use of *P. guajava*. In the acute oral toxicity, no abnormality or mortality was observed in rats at the dose of 5000 mg/kg b.w.; thus, the LD_50_ of the *P. guajava* methanolic bark extract is greater than 5000 mg/kg. This extract is therefore relatively nontoxic since substances with LD_50_ between 2000 mg/kg and 5000 mg/kg orally are considered to have low toxicity, but they, under certain conditions, can be dangerous for vulnerable populations [18]. This study goes in the same direction as that conducted by many researchers such as Sekhar et al. [21] who showed that the *P. guajava* aqueous bark extract has an LD_50_ greater than 5000 mg/kg and that animals remain healthy after 10 days of additional observation. Roy et al. [10] showed that the *P. guajava* leaf extract administered at a dose of 2000 mg/kg has an LD_50_ greater than this dose. Atik et al. [22] showed that the *P. guajava* ethanolic fruit extract administered in mice has an LD_50_ greater than 5000 mg/kg b.w.

In order to evaluate the effects that could result from repeated use of this plant extract, its subacute toxicity was carried out. It should be noted that body weight is an important parameter that can control the health status of an animal; weight loss is frequently used as the first indicator of the harmful effects of drugs [22]. A substance is considered toxic if it causes a mass reduction of more than 10%, and this condition may be considered a sign of toxicity even if other changes do not occur [23]. Animals' weight loss during subacute toxicity can be attributed to antinutritional substances such as tannins and saponins present in this plant. These substances have been reported to cause malabsorption of nutrients in the organism [24]. These antinutrients would be responsible for the low intake of food and thus the reduction in body weight of animals treated at different doses of the extract compared to controls. These results corroborate those of Onyekwe et al. [25] who showed progressive weight loss in animals treated with the ethanolic extract of *P. guajava* roots for 90 days. However, in this study, the weight loss is less than 2%, showing that this plant was not toxic.

The evaluation of hematological parameters provides valuable information on the side effects of foreign substances with respect to the hematopoietic system [26] because this system is one of the most susceptible targets for toxic compounds, especially in the bone marrow where red blood cells are produced [27]. The increased amount of red blood cells in females and platelets in males at 1000 mg/kg b.w. indicates that this plant could possess antianemia property [28] and immunomodulatory effect [29]. Although the specific mechanism through which this extract increases these hematological indices was not ascertained in this study, this effect is assumed to be a direct action of the extract on the hematopoietic system. The nonsignificant difference of the other parameters (hemoglobin, hematocrit, etc.) with respect to the control indicates that the blood elements have not been damaged and that the blood's ability to transport oxygen to the tissues has been preserved during this study. This extract therefore does not affect the hematopoietic system. Uboh et al. [30] have shown that the aqueous *P. guajava* leaf extract administered in male and female rats for 30 days has a hematopoietic potential. The increased amount of white blood cells observed in this study at 1000 mg/kg b.w. in females could therefore explain the inflammation of the liver shown by histological analysis.

Transaminases are enzymatic biomarkers that can indicate tissue damage caused by chemical compounds before structural damage could be observed by conventional histological techniques [31]. These enzymes are synthesized in the cytoplasm and released into the general circulation when the cells are damaged [32] and at some level can be used to verify the extent of hepatocellular damage [33]. In females, the extract selectively affected transaminases; the AST level increased significantly, while the ALT level of the treated animals was not significantly different from that of controls. Since ALT activity is more sensitive to hepatocyte integrity than that of AST [34], this increase in the serum level of AST may be due to the alteration of another organ. However in males, the decrease in ALT and AST levels suggests that this plant extract acts on the liver. Investigations carried by Uboh et al. [30] showed a decrease in transaminase levels in males treated with the aqueous leaf extract of *P. guajava* and an increase in the AST level in females; they also reported a nonsignificant effect of the aqueous extract of *P. guajava* leaves on the liver function enzymes and the morphological architecture of the liver tissues. These results reinforce the hypothesis that the inflammation observed in our study is not related to the increase in the serum level of AST. This decrease in transaminase levels may also reflect the hepatoprotective effect of this plant extract. The work of Roy et al. [10] has also shown the hepatoprotective effect of the aqueous extract of *P. guajava* leaves, with a more pronounced effect at a dose of 500 mg/kg b.w. in both sexes.

The liver and kidney are target organs of toxic chemical compounds because of their essential functions in the processes of detoxification and excretion; these organs are very useful in toxicity studies because of their sensitivity to dangerous compounds [35]. In this work, the indices that assessed renal function were creatinine and urea levels. The kidney regulates the excretion of urea and the reabsorption of electrolytes into the blood; when impaired glomerular function occurs, normally excreted substances such as urea and creatinine accumulate in body fluids [29]. Abnormally high serum levels of creatinine, uric acid, and urea are biomarkers of possible kidney malfunction [36]. In our study, although serum levels of urea and creatinine increased, the urinary levels of these two parameters are normal compared to those in the control. Moreover, the sections of the animal's kidneys showed no alteration of this organ, indicating that the kidneys play their excretory role well. The increase in the serum urea level following administration of the methanol extract of *P. guajava* may be due to increased protein catabolism. Hence, the relative weight of the kidney could be explained by the hyperactivity of the kidney to detoxify the body.

The decrease in the level of serum proteins observed in the animals treated at doses 500 and 1000 mg/kg b.w. and the increase in the urinary protein level at these same doses suggest that the extract would have interfered with the synthesis balance and degradation of total proteins; such decreases can result in hydration that is detrimental to cellular homeostasis.

The increase in total cholesterol and LDL cholesterol and the decrease in HDL cholesterol and triglycerides observed in female rats are an indication of a disturbance of lipid metabolism following the administration of this extract to animals. The *P. guajava* methanolic bark extract could therefore have hyperlipidemic property. This disturbance could lead to risks of cardiovascular diseases because LDL is responsible for the development of fatty deposits and atheromatous plaques on the arteries [37].

## 5. Conclusion

This study provides information on the toxicological profile of the methanol extract of the *Psidium guajava* bark; the results obtained demonstrate that the single dose of 5000 mg/kg b.w. of this plant extract administered orally is not toxic. After long-term treatment (28 days) at high doses (1000 mg/kg b.w.), the toxic effects observed were sex specific and the plant may have some hematological potency and hepatoprotective activity with mild organ toxicity.

## Figures and Tables

**Table 1:** Body weight of animals during acute toxicity.

Animals	Initial weight (g)	Final weight (g)	Weight gain (g)
01	197	198	1
02	188	190	2
03	189	190	1

**Table 2:** Effect of oral administration of the *Psidium guajava* extract on the relative weight of organs and weight gain.

Parameters	Females				Males			
	Control	250 mg/kg	500 mg/kg	1000 mg/kg	Control	250 mg/kg	500 mg/kg	1000 mg/kg
Liver	3.364 ± 0.077^c^	2.928 ± 0.026^b^	2.962 ± 0.037^b^	2.842 ± 0.001^a^	3.276 ± 0.133^c^	2.770 ± 0.049^b^	2.491 ± 0.002^a^	2.694 ± 0.057^b^
Kidney	0.573 ± 0.007^a^	0.612 ± 0.001^b^	0.610 ± 0.005^b^	0.662 ± 0.0005^c^	0.632 ± 0.015^a^	0.633 ± 0.002^a^	0.633 ± 0.001^a^	0.630 ± 0.007^a^
Heart	0.301 ± 0.004^a^	0.362 ± 0.0006^c^	0.355 ± 0.002^c^	0.326 ± 0.007^b^	0.324 ± 0.002^c^	0.320 ± 0.006^c^	0.271 ± 0.006^a^	0.285 ± 0.002^b^
Lung	0.534 ± 0.002^a^	0.666 ± 0.005^b^	0.710 ± 0.003^d^	0.700 ± 0.005^c^	0.725 ± 0.010^d^	0.593 ± 0.022^b^	0.546 ± 0.003^a^	0.637 ± 0.0009^c^
Spleen	0.472 ± 0.006^d^	0.341 ± 0.007^a^	0.370 ± 0.017^b^	0.404 ± 0.005^c^	0.509 ± 0.038^c^	0.463 ± 0.006^b^	0.397 ± 0.005^a^	0.374 ± 0.001^a^
Weight gain (g)	65.2	−2.9	1.5	−2.7	60.1	12.5	26.5	19.2

Data are expressed as mean ± standard deviation. *n* = 8 (4/sex); the values in the same line for a given sex followed by different letters in exponent are significantly different (*p* < 0.05) according to the Newman–Keuls multiple comparison test.

**Table 3:** Effect of oral administration of the *Psidium guajava* extract on hematological parameters.

Parameters	Females				Males			
	Control	250 mg/kg	500 mg/kg	1000 mg/kg	Control	250 mg/kg	500 mg/kg	1000 mg/kg
WBC (10^3^/*μ*L)	7.05 ± 1.17^a^	9.16 ± 1.63^a^	6.36 ± 1.38^a^	10.05 ± 2.98^a^	5.30 ± 2.21^a^	6.40 ± 1.25^a^	9.46 ± 0.93^a^	7.95 ± 2.67^a^
Lymphocytes (%)	41.58 ± 3.03^a^	34.25 ± 5.85^a^	36.23 ± 0.97^a^	36.85 ± 2.52^a^	34.40 ± 5.65^a^	37.42 ± 3.27^a^	36.17 ± 3.87^a^	38.72 ± 1.53^a^
Monocytes (%)	7.63 ± 1.92^a^	7.76 ± 1.65^a^	7.13 ± 0.30^a^	11.30 ± 2.43^a^	8.36 ± 1.57^a^	6.36 ± 0.76^a^	8.20 ± 3.47^a^	9.23 ± 3.75^a^
Granulocytes (%)	9.20 ± 4.18^a^	23.73 ± 10.08^a^	20.40 ± 2.21^a^	15.00 ± 3.00^a^	23.10 ± 9.80^a^	18.80 ± 6.90^a^	19.47 ± 4.32^a^	13.33 ± 1.90^a^
PLT (10^3^/*μ*L)	677.3 ± 30.07^b^	735.7 ± 16.50^b^	751.0 ± 9.539^b^	494.0 ± 76.08^a^	396.0 ± 10.00^a^	438.0 ± 47.84^a^	529.7 ± 30.09^a^	691.0 ± 108.5^b^
MPV (fL)	11.83 ± 1.12^a^	10.87 ± 1.79^a^	10.93 ± 2.12^a^	9.46 ± 0.75^a^	9.83 ± 1.55^a^	9.13 ± 0.37^a^	8.80 ± 0.70^a^	11.00 ± 1.40^a^
RBC (10^6^/*μ*L)	7.29 ± 0.20^a^	7.32 ± 0.45^a^	7.05 ± 0.09^a^	8.08 ± 0.24^b^	6.34 ± 1.93^a^	8.07 ± 0.30^a^	7.89 ± 0.52^a^	7.67 ± 0.24^a^
HGB (g/dL)	16.20 ± 0.10^a^	16.40 ± 0.90^a^	15.85 ± 0.05^a^	16.10 ± 0.40^a^	16.25 ± 0.05^a^	17.07 ± 0.61^a^	17.05 ± 0.15^a^	17.00 ± 0.20^a^
HCT (%)	43.50 ± 1.00^a^	44.45 ± 2.45^a^	41.50 ± 1.31^a^	45.20 ± 2.10^a^	43.25 ± 0.95^a^	47.03 ± 0.80^a^	45.53 ± 1.88^a^	45.07 ± 4.53^a^
MCV (fL)	62.60 ± 3.98^a^	57.73 ± 2.80^a^	58.93 ± 1.06^a^	55.90 ± 3.89^a^	58.00 ± 0.50^a^	58.37 ± 1.41^a^	60.15 ± 0.55^a^	58.67 ± 4.10^a^
MCH (pg)	22.70 ± 0.88^a^	21.50 ± 0.50^a^	21.87 ± 0.66^a^	20.33 ± 1.42^a^	21.75 ± 0.35^a^	21.13 ± 1.29^a^	21.20 ± 0.81^a^	21.53 ± 0.80^a^
MCHC (g/dL)	37.20 ± 1.10^a^	37.40 ± 0.95^a^	37.37 ± 0.66^a^	36.43 ± 1.65^a^	37.55 ± 0.95^a^	36.27 ± 1.80^a^	36.63 ± 1.01^a^	36.90 ± 3.57^a^

WBC: white blood cells; PLT: platelets; MPV: mean platelet volume; RBC: red blood cells; HGB: hemoglobin; HCT: hematocrit; MCV: mean corpuscular volume; MCH: mean corpuscular hemoglobin; MCHC: mean corpuscular hemoglobin concentration. Data are expressed as mean ± standard deviation. *n* = 6 (3/sex); the values in the same line for a given sex followed by different letters in exponent are significantly different (*p* < 0.05) according to the Newman–Keuls multiple comparison test.

**Table 4:** Effect of oral administration of the *Psidium guajava* extract on urinary biochemical parameters.

Parameters	Females				Males			
	Control	250 mg/kg	500 mg/kg	1000 mg/kg	Control	250 mg/kg	500 mg/kg	1000 mg/kg
Creatinine (mg/dL)	53.07 ± 0.832^c^	42.00 ± 0.400^a^	38.80 ± 3.555^a^	48.53 ± 1.007^b^	64.13 ± 3.449^a^	63.87 ± 1.890^a^	63.87 ± 2.663^a^	58.40 ± 6.053^a^
Urea (mg/dL)	1921 ± 184.2^ab^	1618 ± 197.4^a^	1877 ± 84.59^ab^	2066 ± 118.4^b^	1579 ± 105.3^a^	1482 ± 99.63^a^	4355 ± 171.1^c^	2649 ± 30.39^b^
Proteins (g/dL)	0.2721 ± 0.042^a^	0.1837 ± 0.020^a^	0.7211 ± 0.135^b^	1.020 ± 0.040^c^	1.136 ± 0.112^a^	1.252 ± 0.023^ab^	1.306 ± 0.035^b^	1.395 ± 0.042^b^

Data are expressed as mean ± standard deviation. *n* = 8 (4/sex); the values in the same line for a given sex followed by different letters in exponent are significantly different (*p* < 0.05) according to the Newman–Keuls multiple comparison test.

**Table 5:** Effect of oral administration of the *Psidium guajava* extract on serum biochemical parameters.

Parameters	Females				Males			
	Control	250 mg/kg	500 mg/kg	1000 mg/kg	Control	250 mg/kg	500 mg/kg	1000 mg/kg
ALT (U/I)	66.20 ± 1.968^a^	64.75 ± 2.404^a^	70.36 ± 5.099^a^	68.33 ± 1.758^a^	89.57 ± 2.362^c^	74.21 ± 3.148^b^	42.49 ± 5.613^a^	41.47 ± 3.573^a^
AST (U/I)	46.27 ± 2.310^a^	71.88 ± 1.817^c^	64.02 ± 2.667^b^	58.46 ± 5.535^b^	79.44 ± 0.873^c^	66.06 ± 2.197^a^	69.84 ± 1.155^b^	65.85 ± 1.671^a^
Total cholesterol (mg/dL)	47.14 ± 2.277^a^	62.99 ± 2.364^c^	61.27 ± 0.735^c^	52.70 ± 1.767^b^	90.69 ± 2.414^d^	64.79 ± 4.614^b^	53.19 ± 1.068^a^	70.51 ± 1.258^c^
HDL cholesterol (mg/dL)	35.72 ± 0.705^b^	34.88 ± 5.009^b^	26.57 ± 1.301^a^	21.04 ± 3.239^a^	31.74 ± 2.260^a^	42.24 ± 4.495^b^	49.80 ± 4.061^c^	56.92 ± 1.596^d^
LDL cholesterol (mg/dL)	22.25 ± 2.873^a^	38.32 ± 7.160^b^	44.58 ± 0.7714^b^	40.99 ± 4.274^b^	69.70 ± 0.777^d^	32.84 ± 6.584^c^	14.16 ± 4.669^a^	24.42 ± 0.809^b^
Triglycerides (mg/dL)	54.14 ± 0.885^c^	51.02 ± 1.113^b^	49.37 ± 0.692^b^	46.71 ± 1.113^a^	53.77 ± 0.692^a^	51.48 ± 1.262^a^	53.87 ± 1.301^a^	54.14 ± 1.113^a^
Creatinine (mg/dL)	1.036 ± 0.045^c^	0.571 ± 0.054^b^	0.631 ± 0.045^b^	0.488 ± 0.023^a^	0.4762 ± 0.038^a^	0.7738 ± 0.045^b^	0.8929 ± 0.071^c^	1.048 ± 0.086^d^
Urea (mg/dL)	69.91 ± 4.243^a^	82.41 ± 6.263^b^	90.28 ± 1.389^b^	84.26 ± 1.604^b^	70.83 ± 1.389^a^	74.07 ± 5.782^a^	78.70 ± 2.122^a^	89.81 ± 5.613^b^
Total proteins (g/dL)	4.926 ± 0.071^c^	4.192 ± 0.112^b^	4.072 ± 0.124^b^	3.501 ± 0.023^a^	3.426 ± 0.141^b^	3.401 ± 0.117^b^	3.015 ± 0.069^a^	2.878 ± 0.118^a^

ALT: alanine aminotransferase; AST: aspartate aminotransferase. Data are expressed as mean ± standard deviation. *n* = 8 (4/sex); the values in the same line for a given sex followed by different letters in exponent are significantly different (*p* < 0.05) according to the Newman–Keuls multiple comparison test.

## Data Availability

All data generated or analyzed during this study are included in this published article.
